# Spectral Kurtosis Entropy and Weighted SaE-ELM for Bogie Fault Diagnosis under Variable Conditions

**DOI:** 10.3390/s18061705

**Published:** 2018-05-24

**Authors:** Zhipeng Wang, Limin Jia, Linlin Kou, Yong Qin

**Affiliations:** 1 State Key Lab of Rail Traffic Control and Safety, Beijing Jiaotong University, Beijing 100044, China; lmjia@bjtu.edu.cn (L.J.); 13114254@bjtu.edu.cn (L.K.); yqin@bjtu.edu.cn (Y.Q.); 2 National Engineering Laboratory for System Safety and Operation Assurance of Urban Rail Transit, Guangzhou 510000, China; 3 Beijing Research Center of Urban Traffic Information Sensing and Service Technologies, Beijing Jiaotong University, Beijing 100044, China

**Keywords:** bogie fault diagnosis, spectral kurtosis entropy, weighted self-adaptive evolutionary extreme learning machine, protrugram, variational mode decomposition

## Abstract

Bogies are crucial for the safe operation of rail transit systems and usually work under uncertain and variable operating conditions. However, the diagnosis of bogie faults under variable conditions has barely been discussed until now. Thus, it is valuable to develop effective methods to deal with variable conditions. Besides, considering that the normal data for training are much more than the faulty data in practice, there is another problem in that only a small amount of data is available that includes faults. Concerning these issues, this paper proposes two new algorithms: (1) A novel feature parameter named spectral kurtosis entropy (SKE) is proposed based on the protrugram. The SKE not only avoids the manual post-processing of the protrugram but also has strong robustness to the operating conditions and parameter configurations, which have been validated by a simulation experiment in this paper. In this paper, the SKE, in conjunction with variational mode decomposition (VMD), is employed for feature extraction under variable conditions. (2) A new learning algorithm named weighted self-adaptive evolutionary extreme learning machine (WSaE-ELM) is proposed. WSaE-ELM gives each sample an extra sample weight to rebalance the training data and optimizes these weights along with the parameters of hidden neurons by means of the self-adaptive differential evolution algorithm. Finally, the hybrid method based on VMD, SKE, and WSaE-ELM is verified by using the vibration signals gathered from real bogies with speed variations. It is demonstrated that the proposed method of bogie fault diagnosis outperforms the conventional methods by up to 4.42% and 6.22%, respectively, in percentages of accuracy under variable conditions.

## 1. Introduction

Urban rail transit systems are critical for large modern cities. With the development of urban rail traffic, more and more metro trains are being placed into service, and the safe and reliable operation of these trains has become a hot topic. Bogies, as crucial parts of metro trains, have a great influence on the safe utilization of urban rail traffic [1]. Therefore, many studies have been done on bogie fault diagnosis. Chudzikiewicz et al. [2,3] utilized acceleration signals on wheelset axle-boxes to monitor trains and tracks simultaneously. Qin et al. employed a wavelet feature for bogie fault signal analysis [4]. Cai et al. [5] and Trilla et al. [6] utilized empirical mode decomposition (EMD) for fault diagnosis of railway axle bearings. Na et al. [7] utilized ensemble EMD and manifold learning for bogie fault identification. Bustos et al. [8] utilized EMD for the identification of the bogie operating state of a high-speed train in service. However, all of the existing methods require mainly stable operating conditions. In the real world, bogies usually work under variable and fluctuant conditions. Therefore, it is critical to study the diagnosis of bogie faults under variable conditions.

Due to the joint influence of the train and the track, the vibrations of bogies have remarkable characteristics of nonlinear vibration. Considering the nonlinear signals and heavy background noises, traditional signal analysis algorithms such as Fourier transform (FT) are futile in this situation. To solve this problem, EMD has been applied [5,7]. However, EMD has a significant drawback: mode mixing, which restricts the application of EMD in practice. In 2014, Dragomiretskiy et al. [9] proposed a novel adaptive method called variational mode decomposition (VMD), which can decompose a signal into an ensemble of band-limited intrinsic mode functions (IMFs), each with a center frequency. VMD is an entirely non-recursive and quasi-orthogonal method and has been applied to rotating machinery fault diagnosis [10]. It has been proven that VMD is more efficient than EMD. Therefore, VMD has been employed in this paper for nonlinear signal processing.

As the dynamic relationships of bogies are extremely sophisticated, it is sufficiently challenging to extract features of early faults from vibration signals with a low signal-to-noise ratio (SNR). Spectral kurtosis (SK), proposed by Dwyer [11], can indicate how the impulsiveness of a signal varies with frequency and can detect fault-induced peaks (or protrusions). Antoni further analyzed the concept and proposed the fast kurtogram (FK) [12], which was proven to be effective in early fault diagnosis [13,14,15,16]. However, FK has several shortcomings, especially when the noise is strong and contains high peaks [17]. Thus, a novel method called a protrugram was proposed [17]. A protrugram is calculated by using the kurtosis of envelope spectrum amplitudes of signals and is more effective than FK for detecting transients with low SNR. However, it should predetermine the bandwidth with a priori knowledge and requires manual post-processing to reject discrete tones [17]. Due to the complicated kinematics and variable conditions of bogies, these drawbacks limit their application in bogie fault diagnosis.

In this paper, inspired by the Shannon entropy, a novel feature extraction method named spectral kurtosis entropy (SKE) is proposed to extract fault characteristics under variable conditions. This method calculates the entropy of spectral kurtosis based on the protrugram. As the Shannon entropy can be used to measure the uncertainty of information, spectral kurtosis entropy has a strong robustness to the predetermined bandwidth value and the operating condition and avoids the manual post-processing of the protrugram. In the following study, the advantages of SKE will be verified. Thus, this paper employs SKE for feature extraction under variable conditions.

After feature extraction, the main part is fault classification with the problem of imbalanced data. Extreme learning machine (ELM), as a new effective learning method, has been applied in many studies and has shown great efficiency [18,19,20]. However, since the parameters of hidden neurons in ELM are randomly assigned and invariant in the training processing, a number of useless and non-optimal neurons exist in the trained model. To solve this problem, the self-adaptive evolutionary extreme learning machine (SaE-ELM) was proposed [21,22] and has been proven to outperform the conventional ELM. However, since in practice the normal samples acquired from bogies are much more than the faulty ones, bogie fault diagnosis algorithms suffer from the imbalance of data. To deal with this issue, this paper proposes a novel algorithm named weighted self-adaptive evolutionary extreme learning machine (WSaE-ELM). This method gives each sample an optimizable sample weight to rebalance the training data and employs self-adaptive differential evolution algorithm to optimize these weights and the parameters of the hidden neurons. The proposed WSaE-ELM was employed for bogie fault diagnosis and its feasibility and effectivity was verified in the following study.

This paper is organized as follows: Section 2 introduces VMD, spectral kurtosis entropy, WSaE-ELM, and the scheme of the hybrid method for bogie fault diagnosis under variable conditions; Section 3 describes the simulation verification of VMD and SKE; Section 4 describes the application of the proposed method on bogie fault diagnosis under variable conditions; and Section 5 concludes this paper. The acronyms used in this paper are listed in Table 1.

## 2. Methodology

### 2.1. Variational Mode Decomposition

To decompose a nonlinear signal with low RMSE into a discrete number of intrinsic mode functions (IMFs), VMD assumes that each IMF is around a center frequency and the bandwidth is assigned as its sparsity prior [9]. This method is a non-recursive algorithm and can extract IMFs concurrently. It involves three essential concepts: Wiener filtering, Hilbert transform, and frequency mixing. The details about VMD can be found in Reference [9]. By using VMD, a signal can be decomposed as follows:

(1) $$x(t)=\sum_{i=1}^{k}imf_{i}(t)$$

Here, x(t) is the raw signal and $\left\{imf_{i}\}=\{imf_{1},\ldots ,imf_{k}\right\}$ represents the obtained modes. VMD has shown its great efficiency and strong robustness to sampling and noise [9,10]. In this paper, it is employed for bogie signal processing.

### 2.2. Spectral Kurtosis Entropy

Kurtosis is a dimensionless parameter indicating the impulsiveness (or the protrusion) of a signal. The definition is as follows:

(2) $$K=\frac{E{\left(X-\mu \right)}^{4}}{\sigma^{4}}$$

Here, X is the signal, μ and σ are the mean value and standard deviation of the signal, respectively. For a vector $X=\{x_{1},x_{2},\cdots ,x_{N}\}$, Equation (2) can be illustrated as follows:

(3) $$K(X)=\frac{E{\left(X-\mu \right)}^{4}}{\sigma^{4}}=\frac{(1/N)\sum_{i=1}^{N}{\left(x_{i}-\mu \right)}^{4}}{{\left(\sqrt{(1/N)\sum_{i=1}^{N}{\left(x_{i}-\mu \right)}^{2}}\right)}^{4}}=\frac{(1/N)\sum_{i=1}^{N}{\left(x_{i}-\mu \right)}^{4}}{{\left((1/N)\sum_{i=1}^{N}{\left(x_{i}-\mu \right)}^{2}\right)}^{2}}=\frac{(1/N)\sum_{i=1}^{N}{\left[x_{i}-\left((1/N)\sum_{i=1}^{N}x_{i}\right)\right]}^{4}}{{\left\{(1/N)\sum_{i=1}^{N}{\left[x_{i}-\left((1/N)\sum_{i=1}^{N}x_{i}\right)\right]}^{2}\right\}}^{2}}$$

Here, N is the number of sample points of the signal X. Kurtosis has been applied widely on mechanical fault diagnosis but it is a time-domain parameter and cannot extract transients of the signal.

**Figure 1:**
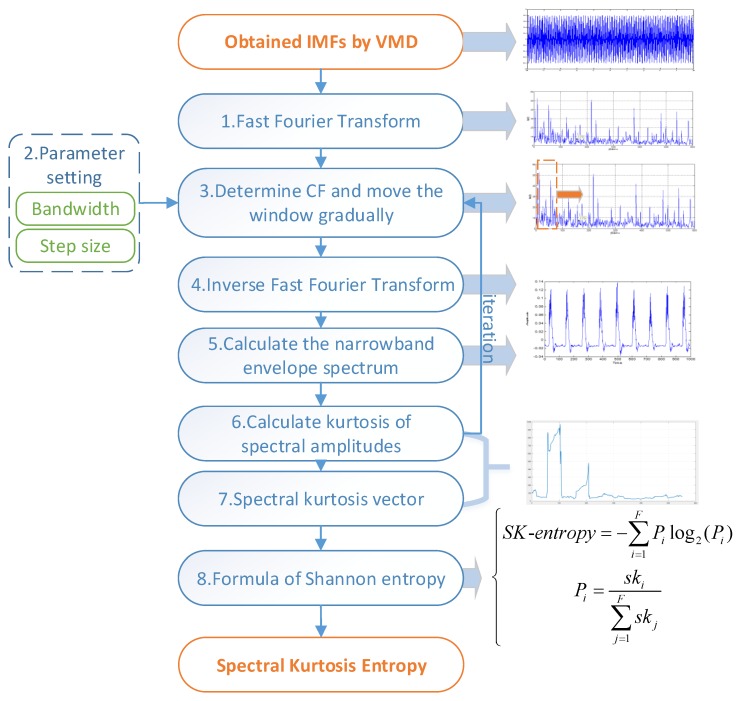
The procedure of spectral kurtosis entropy.

The protrugram is a kurtosis-based algorithm [17] and computes spectral kurtosis values by using the amplitudes of the spectrum of the narrowband envelopes of the signal. Compared with FK, the protrugram should predetermine the bandwidth (BW) and seek the optimal center frequency (CF). The setting of the BW depends mainly on expertise and a priori knowledge. Besides, additional manual post-processing is also essential for the protrugram. To deal with these drawbacks and automatically extract fault characteristics under variable conditions, this paper proposed a novel feature parameter named spectral kurtosis entropy by means of the Shannon entropy theory. As shown in Figure 1, the steps of spectral kurtosis entropy are as follows:

(1) Based on the obtained IMFs by VMD, the fast Fourier transform is calculated to acquire frequency-domain results of IMFs.

(2) The BW and step size are assigned to fixed values. The value of BW used to be approximately three to five times larger than the fault eigenfrequency. In the following study, the proposed SKE has been proven to be insensitive to the parameter setting.

(3) The CF is assigned numbers ranging from BW/2 to f/2 gradually (f is the sampling frequency), and the corresponding window is determined.

(4) Inverse fast Fourier transform is employed to process the narrowband signal.

(5) The narrowband envelope spectrum is calculated.

(6) The kurtosis of spectral amplitudes of positive frequencies is computed.

(7) Steps 3 to 6 are repeated until the spectral kurtosis vector $SK=\{sk_{1},sk_{2},\cdots ,sk_{F}\}$ is acquired.

(8) According to the definition of spectral kurtosis entropy, its value is calculated. The equations are as follows:

(4) $$\{\begin{array}{c} SK-entropy=-\sum_{i=1}^{F}P_{i}\log_{2}(P_{i})\\ P_{i}=\frac{sk_{i}}{\sum_{j=1}^{F}sk_{j}} \end{array}$$

Here, SK-entropy is the spectral kurtosis entropy, $sk_{i}$ is from the spectral kurtosis vector $SK=\{sk_{1},sk_{2},\cdots ,sk_{F}\}$, F is the length of SK, and *i* varies from 1 to F.

In the following study, the proposed SKE has been proven to have strong robustness to the setting of the BW and also avoids the manual post-processing. Therefore, it can self-adaptively extract fault features under variable conditions.

### 2.3. Weighted Self-Adaptive Evolutionary ELM

**Figure 2:**
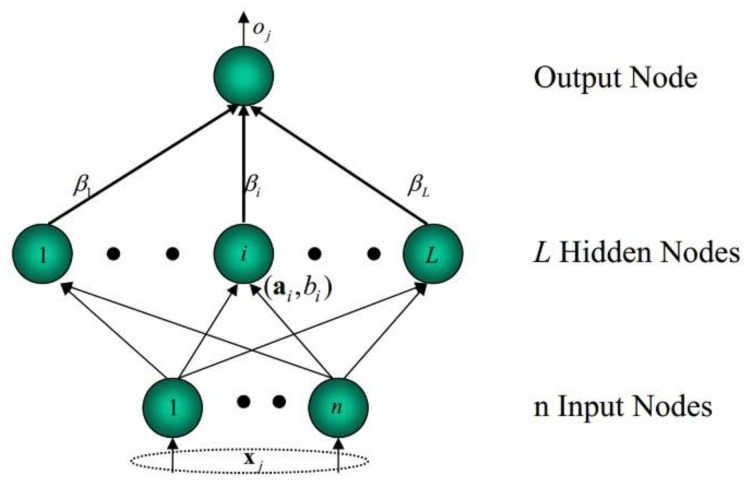
The structure of the extreme learning machine (ELM).

ELM was proposed for single-hidden-layer feedforward networks (SLFNs) and has been widely studied and applied due to its remarkable performance [23,24,25]. In ELM, the input weights and biases of hidden neurons are randomly allocated with no adjusting during the training, and the output weights are calculated efficiently using a least square algorithm. The structure is shown in Figure 2. Details can be found in Huang et al. [23].

For an arbitrary training dataset $\left(x_{i},y_{j}),\;(i=1,2,\cdots ,N;\;j=1,2,\cdots ,M\right)$, the trained model of ELM with *L* hidden neurons can be described as follows:

(5) $$o_{i}(x)=\sum_{i=1}^{L}\beta_{i}g\left(\alpha_{i},b_{i},x\right)=H\cdot \beta$$

Here, $\alpha_{i}$, $b_{i}$ and $\beta_{i}$ are the input weight vector, bias, and output weight vector of the *i*th hidden neuron, respectively; g(·) is the selected activation function. H is the output matrix of the hidden layer and can be described as

(6) $$H={\left[\begin{array}{ccc} g(\alpha_{1},b_{1},x_{1}) & \cdots & g(\alpha_{L},b_{L},x_{1})\\ \vdots & \ddots & \vdots \\ g(\alpha_{1},b_{1},x_{N}) & \cdots & g(\alpha_{L},b_{L},x_{N}) \end{array}\right]}_{N\times L}={\left[\begin{array}{ccc} g(\alpha_{1}\cdot x_{1}+b_{1}) & \cdots & g(\alpha_{L}\cdot x_{1}+b_{L})\\ \vdots & \ddots & \vdots \\ g(\alpha_{1}\cdot x_{N}+b_{1}) & \cdots & g(\alpha_{L}\cdot x_{N}+b_{L}) \end{array}\right]}_{N\times L}$$

To deal with the data imbalance, each training sample is given an extra sample weight, $w_{j}(j=1,2,\cdots ,N)$, which is randomly assigned initially and optimized during the training. Thus, the output matrix H can be redefined as follows:

(7) $$H={\left[\begin{array}{ccc} g(\alpha_{1}\cdot w_{1}\cdot x_{1}+b_{1}) & \cdots & g(\alpha_{L}\cdot w_{1}\cdot x_{1}+b_{L})\\ \vdots & \ddots & \vdots \\ g(\alpha_{1}\cdot w_{N}\cdot x_{N}+b_{1}) & \cdots & g(\alpha_{L}\cdot w_{N}\cdot x_{N}+b_{L}) \end{array}\right]}_{N\times L}$$

**Figure 3:**
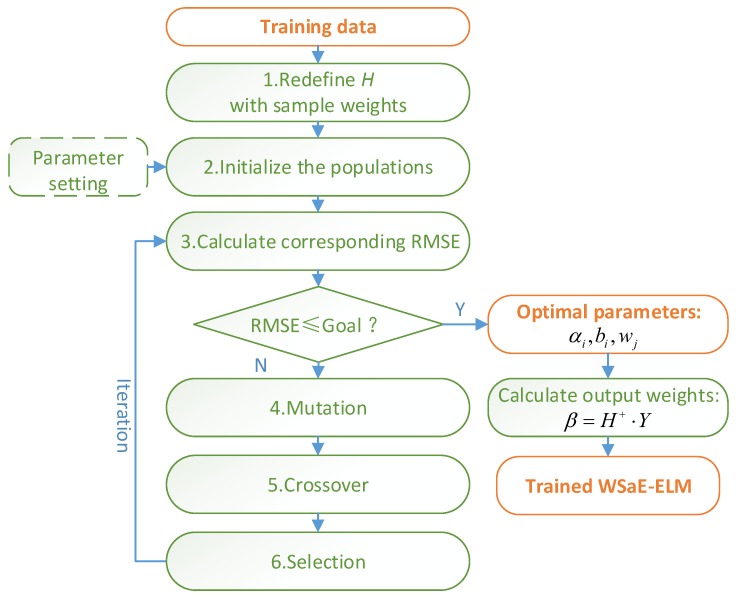
The procedure of weighted self-adaptive evolutionary extreme learning machine (WSaE-ELM).

Since the parameters $\alpha_{i}$ and $b_{i}$ are randomly assigned and remain unchanged, the network generates a lot of non-optimal neurons that are useless for classification. To solve this issue, this study has employed the self-adaptive differential evolutionary algorithm for adaptive optimization of the parameters $\alpha_{i}$, $b_{i}$, $w_{j}$ together. Therefore, a novel algorithm named weighted self-adaptive differential evolutionary ELM (WSaE-ELM) is proposed. As shown in Figure 3, the steps of WSaE-ELM are as follows:

(1) Redefine the output matrix H by introducing sample weights $w_{j}\;(j=1,2,\cdots ,N)$.

(2) Initialize the original populations, which consist of the parameters $\alpha_{i},b_{i},w_{j}$:

(8) $$\theta_{r,G}=\left[\alpha_{1,(r,G)}^{T},\cdots ,\alpha_{L,(r,G)}^{T},b_{1,(r,G)},\cdots ,b_{L,(r,G)},w_{1,(r,G)}^{T},\cdots ,w_{N,(r,G)}^{T}\right]$$

where G is the generation and r=1,2,⋯,NP.

(3) Calculate, according to the least square method, the output weights β and root mean square error (RMSE) corresponding to each population vector:

(9) $$\beta_{r,G}=H_{r,G}^{+}\cdot Y$$

(10) $$RMSE_{r,G}=\sqrt{\frac{\sum_{i=1}^{N}\|\sum_{j=1}^{L}\beta_{j}g(\alpha_{j,(r,G)}\cdot w_{i,(r,G)}\cdot x_{i}+b_{j,(r,G)})-y_{i}}{M\cdot N}}$$

where $H_{r,G}^{+}$ is the Moore–Penrose pseudo inverse of $H_{r,G}^{}$.

(11) $$H_{r,G}={\left[\begin{array}{ccc} g(\alpha_{1,(r,G)}\cdot w_{1,(r,G)}\cdot x_{1}+b_{1,(r,G)}) & \cdots & g(\alpha_{L,(r,G)}\cdot w_{1,(r,G)}\cdot x_{1}+b_{L,(r,G)})\\ \vdots & \ddots & \vdots \\ g(\alpha_{1,(r,G)}\cdot w_{N,(r,G)}\cdot x_{N}+b_{1,(r,G)}) & \cdots & g(\alpha_{L,(r,G)}\cdot w_{N,(r,G)}\cdot x_{N}+b_{L,(r,G)}) \end{array}\right]}_{N\times L}$$

(12) $$\theta_{r,G+1}=\{\begin{array}{cc} u_{r,G+1} & RMSE_{\theta_{r,G}}-RMSE_{u_{r,G+1}}>\lambda \cdot RMSE_{\theta_{r,G}}\\ u_{r,G+1} & \begin{array}{c} \left|RMSE_{\theta_{r,G}}-RMSE_{u_{r,G+1}}\right|<\lambda \cdot RMSE_{\theta_{r,G}}\\ and\left\|\beta_{u_{r,G+1}}\right\|<\left\|\beta_{\theta_{r,G}}\right\| \end{array}\\ \theta_{r,G} & else \end{array}$$

where $\theta_{r,G+1}$ is the (*G* + 1)th candidature vector generated based on RMSE; $u_{r,G+1}$ is the (*G* + 1)th trial vector; and λ is assigned to 0.015 by default.

(4) Obtain the new mutant vectors $v_{r,G}$ by mutation. There are four selectable mutation strategies as shown in Table 2. In this paper, the strategy is selected adaptively based on the probability $p_{s,G}$, which means the probability of selecting the strategy *s* is (*s* = 1,2,3,4). Details can be found in Reference [21]. The factor F is used to determine the step size and obeys the normal distribution; K is randomly assigned [0, 1]. $k_{1}\cdots k_{5}$ are random integers in the range of 1,2,⋯,NP.

(5) After the mutation process, calculate the trial vector $u_{r,G}$ by means of a crossover procedure:

(13) $$u_{{}_{r,G}}^{j}=\{\begin{array}{cc} v_{i,G}^{j} & \left(rand_{j}\leq CR)or(j=j_{rand}\right)\\ \theta_{i,G}^{j} & otherwise \end{array}$$

where CR represents the crossover rate, which can be valued in [0, 1]; $rand_{j}$ is randomly assigned in [0, 1]; $j_{rand}$ is a random integer from 1,2,⋯,NP.

(6) Consider RMSE as the fitness function. When the value of RMSE is the lowest, the corresponding target vector and trial vector are stored for the next population.

(7) Repeat Steps 3 to 6 until the goal is achieved or the maximum iterations are arrived at.

By involving the sample weight, WSaE-ELM assigns a different misclassification cost for each sample to rebalance the data. Compared with the conventional ELM and SaE-ELM, WSaE-ELM not only improves the stability and generalization ability but also overcomes the imbalance of data.

### 2.4. Bogie Fault Diagnosis Based on VMD, SKE and WSaE-ELM

**Figure 4:**
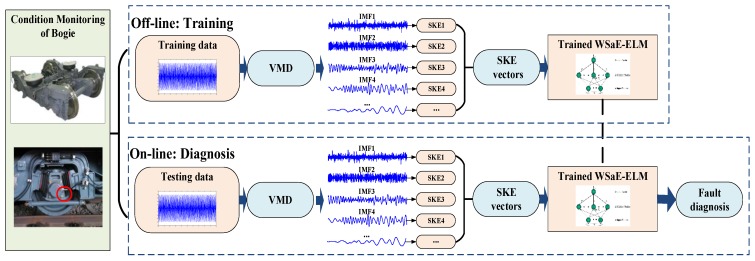
The scheme of the proposed method.

In this study, VMD, SKE, and WSaE-ELM were employed for bogie fault diagnosis under variable conditions. The scheme is illustrated in Figure 4.

(1) Feature extraction. VMD is first applied to decompose the vibration signals of the bogie into *n* IMFs. Then, the spectral kurtosis entropy of each IMF is calculated to form an n-dimensional feature vector. The calculation processes of VMD and SKE can be found in Section 2.1 and Section 2.2, respectively.

(2) Fault classification. Based on the acquired features, the proposed WSaE-ELM is utilized to diagnose bogie faults under variable conditions by using imbalanced training data. More details about the novel WSaE-ELM can be found in Section 2.3.

## 3. Simulation Experiment

### 3.1. Simulation Data

To demonstrate the efficiency of VMD and SKE for feature extraction, a simulated mechanical vibration signal x(t) was involved. Assume that the rotational speed increased from 0 to the rated speed 1800 r/min in the 0–100 s time frame and then remained stable in the 100–120 s time frame. The simulation model can be described as follows:

(14) $$x(t)=\{\begin{array}{cc} x_{1}(t)\cdot \sin\left(x_{2}(t)\cdot t+\frac{\pi }{2}\right) & 0<t\leq 100\\ 200\cdot \sin\left(60\pi \cdot t+\frac{\pi }{2}\right) & 100<t\leq 120 \end{array}$$

(15) $$x_{1}(t)=20\cdot t^{2}$$

(16) $$x_{2}(t)=\frac{3}{10}\pi \cdot t$$

**Figure 5:**
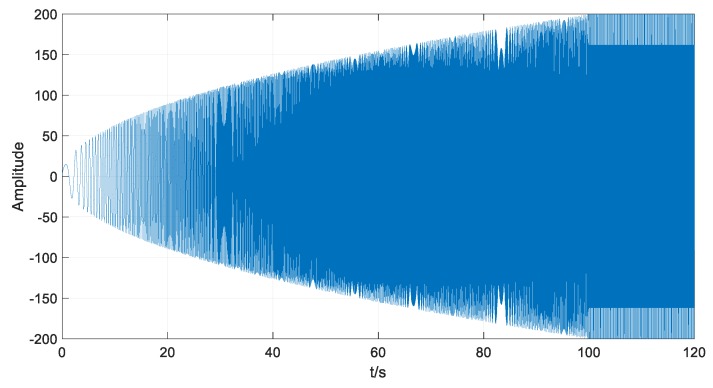
The generated simulated signal.

**Figure 6:**
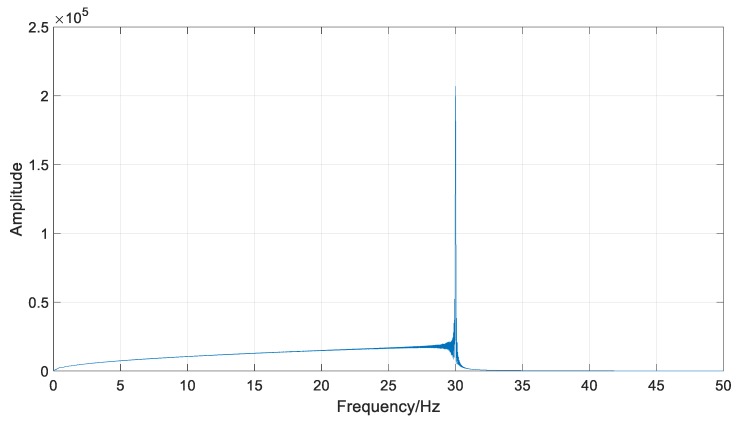
The frequency-domain result of the simulated signal.

The sample rate was set to 100 S/s. The acquired data are shown in Figure 5 and Figure 6.

### 3.2. Simulation Result by Using VMD

**Figure 7:**
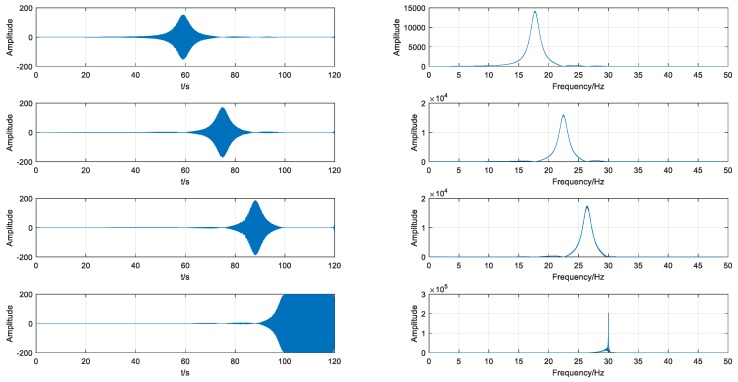
Results of variational mode decomposition (VMD) from the simulated signal.

**Figure 8:**
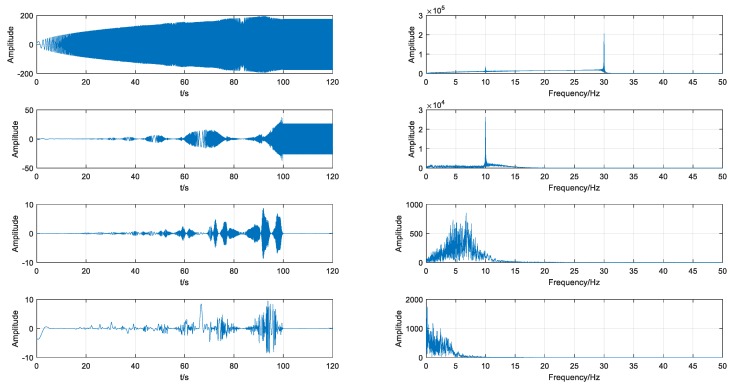
Results of empirical mode decomposition (EMD) from the simulated signal.

After the generation of the simulated signal, VMD was employed to decompose the signal into four IMFs, as shown in Figure 7. For comparison, IMFs by EMD were also calculated, as shown in Figure 8. It is obvious that the IMFs obtained by EMD are chaotic and cannot accurately represent the characteristics of the signal under different operating conditions. On the contrary, each IMF obtained by VMD is able to precisely represent the information of the signal in a certain stage. $IMF_{1},IMF_{2},IMF_{3}$ correspond to different sequential stages under variable conditions and appear to be spindle. $IMF_{4}$ corresponds to the stable stage and the amplitude is almost constant. Compared with EMD, VMD can decompose the signal more effectively, especially under variable operating conditions.

### 3.3. Simulation Result by Using SKE

Based on the IMFs obtained by VMD, the proposed SKE was tested to verify whether it has strong robustness to variable conditions and bandwidth settings. In this study, the bandwidth (BW) was assigned to 5, 7, 10, 13, 15, 17, 20 Hz sequentially.

**Figure 9:**
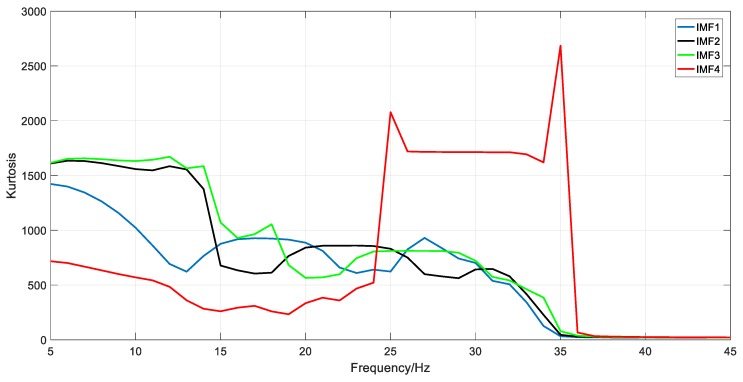
Results of the protrugram when the bandwidth (BW) = 10 Hz.

First, the spectral kurtosis was obtained by means of a protrugram. For instance, when the BW was 10 Hz, the results of the protrugram were calculated and are shown in Figure 9. It is implied that there are notable differences among the spectral kurtosis of different IMFs, especially the $IMF_{4}$, which represents the stable stage. That means the spectral kurtosis is under the influence of the operating condition.

Second, the spectral kurtosis entropies of IMFs were calculated, as shown in Table 3. It can be observed that the SKEs of each IMF are almost identical while the BW varies from 5 to 20 Hz, which means SKE is insensitive to the setting of the BW. Moreover, the SKEs of different IMFs are also roughly similar. As previously mentioned, each IMF represents a stage with a corresponding operating condition. Therefore, it is implied that SKE has a strong robustness to the operating condition and can be employed for feature extraction under variable conditions.

## 4. Case Study

### 4.1. Experimental Setup

**Figure 10:**
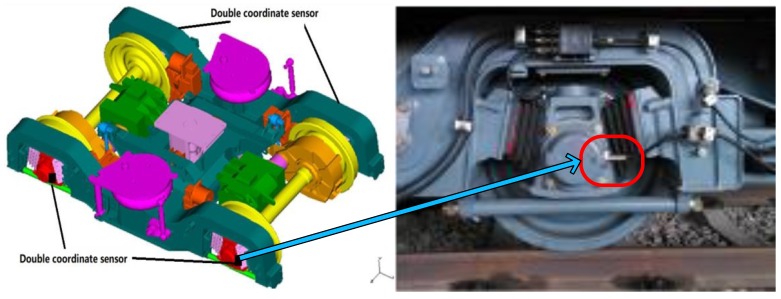
Locations of the accelerometers.

The real vibration signals of bogies were acquired from A-type trains by a Chinese metro company. The weights of the trains involved were roughly 38 tons without load during the experiments. This study utilized a simplified signal acquisition system to obtain the vibration signals of the bogies. There were four accelerometers deployed on each bogie, and the locations are shown in Figure 10. The accelerometers were type 787T and produced by the Wilcoxon Company. The signals were gathered with a sampling rate of 10 kS/s. Then, the access points were utilized to transfer the acquired data to an industrial computer (Advantech TPC-1251H) and the analog-to-digital conversion and digital signal filtering were conducted by signal conditioners. Finally, the industrial computer collected and stored the data.

**Figure 11:**
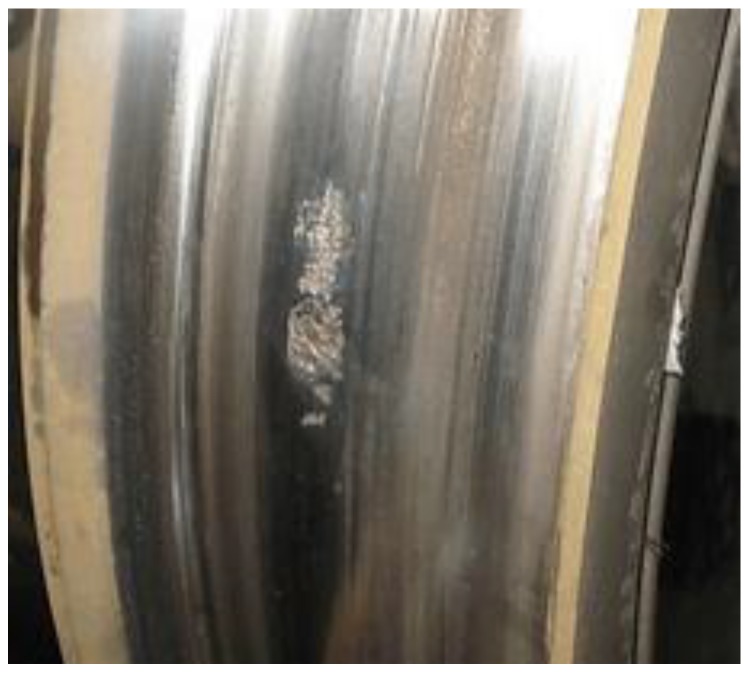
An example of the bogie fault (wheel tread peeling).

**Figure 12:**
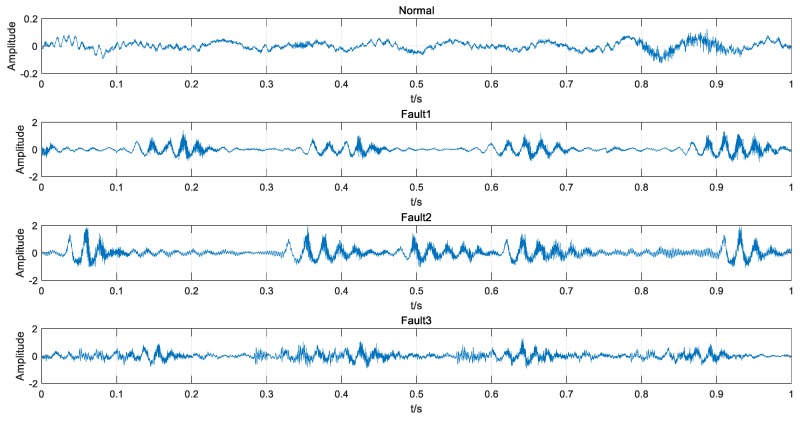
Examples of bogie signals.

The trains were running at a fluctuant speed of 35 ± 10 km/h while gathering the signals. Therefore, all data were acquired under uncertain and variable conditions. The collected data contained four types of bogie conditions: normal condition (Normal), wheel out-of-roundness or tread peeling (Fault1, as shown in Figure 11), axle misalignment (Fault2), wheel runout (Fault3). Each type had a different number of samples, as shown in Table 4 and Figure 12. It is obvious that the imbalanced data problem existed.

### 4.2. Feature Extraction

**Figure 13:**
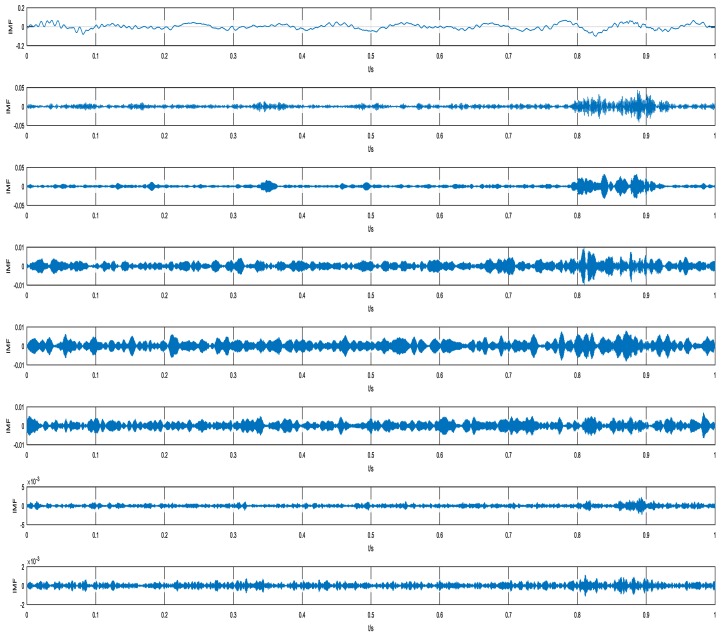
An example of results by VMD under normal conditions.

In the beginning, VMD was utilized to decompose the raw vibration signal into *n* IMFs. Here, *n* was assigned to 8. For instance, the result of a normal signal obtained by VMD is shown in Figure 13. Then, the SKE of each IMF was calculated. In this study, the BW was assigned to 10 Hz by default. Finally, an 8-dimensional feature vector from each sample was obtained.

### 4.3. Fault Classification

On the basis of the features extracted by means of VMD and SKE, the proposed WSaE-ELM was employed for bogie fault diagnosis under variable conditions. For comparison, the conventional ELM, SaE-ELM, and the traditional weighted ELM (WELM) [26] were also utilized for bogie fault classification. The results are illustrated in Table 5.

As shown in Table 5, the total accuracy rates of the four models are all greater than 90%, which implies that the proposed feature parameter SKE is able to precisely extract fault characteristics with little influence of variable conditions. However, although the total accuracy rates of ELM and SaE-ELM seem to reach a satisfactory level, their false negative rates are 26.36% and 20.91%, respectively, which is extremely high in practice. For example, if Fault1 happened, there is only a 63.33% probability of identifying the fault accurately by using ELM. The SaE-ELM performs better than ELM, but still only has an accuracy rate of 70%. Their high false negative rates are unacceptable in practice. Obviously, these are caused by the imbalanced training data. If the training data are imbalanced, the classifiers may tend towards the category with a larger sample size. Since the normal samples for training are much more than the faulty ones, the fault classifiers tend to classify test samples into the normal category. The traditional WELM is able to eliminate the influence of imbalanced data to some extent and reduces the false negative rate to 8.18%. However, weights of the WELM are set a priori according to the number of samples belonging to each class and cannot be optimized during the training process. Therefore, the total accuracy of WELM, which is restricted to the non-optimal sample weights and hidden neurons, has no significant improvement. WSaE-ELM not only gives each training sample an extra weight, but also optimizes these weights as well as the parameters of the hidden neurons. Therefore, WSaE-ELM performed effectively in this study and increased the total accuracy significantly. By using actual data that had been acquired from A-type trains by a Chinese metro company, the WSaE-ELM has been verified as being usable for bogie fault diagnosis with imbalanced data under variable conditions.

## 5. Conclusions

This paper discusses mainly bogie fault diagnosis under variable conditions. To this end, a novel feature parameter SKE was proposed first. The simulation results show that the SKE, in conjunction with VMD, has strong robustness under operating conditions and parameter crations and can be used for feature extraction under variable conditions. Then, a novel learning algorithm WSaE-ELM was proposed for bogie fault classification. WSaE-ELM gives each training sample an optimizable weight to deal with the imbalanced data and utilizes the self-adaptive differential evolution algorithm for parameter optimization. A hybrid method based on VMD, SKE, and WSaE-ELM was implemented for fault diagnosis of real bogies under variable conditions. Compared with the conventional methods, the proposed method increases the accuracy rate by up to 6.22% and reduces the false negative rate to 4.55%. The results demonstrate that the proposed method performs effectively and efficiently in dealing with imbalanced training data under variable conditions.

However, in view of the hardware conditions, this study involved only three typical fault modes of bogies. Besides, the running speed fluctuated only in a narrow region; increased fluctuating conditions might cause additional difficulties. Thus, further work is needed to verify the generality and efficiency of the method.

## Figures and Tables

**Table 1:** The list of acronyms.

Acronym	Full Name
VMD	variational mode decomposition
IMF	intrinsic mode function
SKE	spectral kurtosis entropy
ELM	extreme learning machine
SaE-ELM	self-adaptive evolutionary extreme learning machine
WELM	weighted extreme learning machine
WSaE-ELM	weighted self-adaptive evolutionary extreme learning machine
BW	bandwidth
FK	spectral kurtosis
CF	center frequency
FT	Fourier transform
SNR	signal-to-noise ratio
RMSE	root mean squared error

**Table 2:** Mutation strategies.

Strategy	Expression
DE/rand/1	$v_{r,G}=\theta_{k1,G}+F(\theta_{k2,G}-\theta_{k3,G})$
DE/rand/1-to- best/2	$v_{r,G}=\theta_{k1,G}+F(\theta_{best,G}-\theta_{k1,G})+F(\theta_{k2,G}-\theta_{k3,G})+F(\theta_{k4,G}-\theta_{k5,G})$
DE/rand/2	$v_{r,G}=\theta_{k1,G}+F(\theta_{k2,G}-\theta_{k3,G})+F(\theta_{k4,G}-\theta_{k5,G})$
DE/current-to-rand/1	$v_{r,G}=\theta_{k1,G}+K(\theta_{k1,G}-v_{r,G})+F(\theta_{k2,G}-\theta_{k3,G})$

**Table 3:** The calculated spectral kurtosis entropies.

Signal	BW(Hz)							Mean
	5	7	10	13	15	17	20	
IMF1	4.9846	4.9144	4.8798	4.8779	4.8745	4.8631	4.8472	4.8916
IMF2	4.9693	4.8807	4.8194	4.8092	4.8211	4.8379	4.8740	4.8588
IMF3	4.9974	4.9118	4.8411	4.8197	4.8184	4.8349	4.8898	4.8733
IMF4	4.8032	4.7621	4.75766	4.7565	4.7552	4.7679	4.7992	4.7717

**Table 4:** Datasets of bogie fault diagnosis.

Type	Bogie of A-Type Train				Total
	Normal	Fault1	Fault2	Fault3	
No. of training samples	1500	60	60	300	1920
No. of test samples	500	30	30	50	610
Sum	2000	90	90	350	2530

**Table 5:** Results of bogie fault diagnosis.

	Test Accuracy Rate					False Positive Rate	False Negative Rate
	Normal	Fault1	Fault2	Fault3	Total		
ELM	96.20%	63.33%	70.00%	74.00%	91.48%	3.80%	26.36%
SaE-ELM	96.80%	70.00%	73.33%	82.00%	93.11%	3.20%	20.91%
WELM	94.80%	80.00%	86.67%	90.00%	93.28%	5.20%	8.18%
WSaE-ELM	98.40%	90.00%	96.66%	96.00%	97.70%	1.60%	4.55%
